# Learning well, living well: the causal effects of higher education on self-rated health and mental health in China

**DOI:** 10.3389/fpubh.2025.1636194

**Published:** 2025-08-11

**Authors:** Shudong Yang

**Affiliations:** Party Committee Office, Southeast University, Nanjing, China

**Keywords:** education, public health, China, mental health, self-rated health

## Abstract

**Background:**

In the context of rapid population aging and the global health challenges posed by the COVID-19 pandemic, understanding the social determinants of health has become increasingly important. Education, as a key socioeconomic factor, plays a critical role in shaping individual health outcomes. However, empirical evidence on the causal relationship between higher education (HE) and health, particularly in developing countries like China, remains limited.

**Methods:**

Utilizing data from the 2022 China Family Panel Studies (CFPS), this study primarily employed Ordinary Least Squares (OLS) regression to estimate the effects of HE on self-rated health (SRH) and mental health (MH). To address potential endogeneity and strengthen causal inference, an instrumental variable (IV) approach was implemented. Robustness tests included substituting explanatory variables, excluding specific samples, and employing an ordered Probit model. Mechanism analysis explored the roles of health behaviors, household income, and social capital. Intergenerational effects of parental HE on children’s health were also examined.

**Results:**

Higher education was found to have a statistically significant positive impact on both SRH and MH (*p* < 0.01). These results remained consistent across all robustness tests and when using the IV approach, supporting a causal interpretation. Mediation analysis revealed that HE improves health by fostering healthier behaviors, increasing household income, and enhancing social capital. Furthermore, paternal HE significantly improved children’s SRH (*p* < 0.05), while maternal HE significantly enhanced children’s MH (*p* < 0.01).

**Conclusion:**

This study provides robust evidence that higher education serves as a crucial determinant of health in China, with beneficial effects extending across generations. The findings highlight the potential of educational interventions as a strategic avenue for improving public health and reducing health disparities.

## 1 Introduction

In recent years, global public health has faced unprecedented challenges due to population aging and the COVID-19 pandemic (1), both of which have profoundly impacted physical health and mental health (MH) worldwide (2). Health is a fundamental determinant of human well-being and social development (3). Understanding the factors that influence health outcomes is thus essential for individuals and policymakers seeking to improve public health and enhance economic productivity (4). Among the various determinants of health, access to healthcare services and medical technologies has traditionally been regarded as a key driver (5). However, increasing attention has been paid to the role of health literacy and the adoption of healthy behaviors in shaping long-term health outcomes (6). Higher education (HE), as a crucial stage of human capital formation and a distinct educational setting, not only contributes to the acquisition of knowledge and skills but also fosters cognitive abilities, critical thinking, and decision-making capacities that directly influence health-related behaviors and choices (7). A growing body of literature suggests that individuals with HE tend to experience better employment prospects (8), higher income (9), improved social status (10), and enhanced quality of life (11). Despite these widely acknowledged benefits, whether and how HE exerts a lasting influence on health remains an important but insufficiently explored question.

Within health economics, a substantial body of literature has established a positive education-health gradient, primarily in developed countries. This gradient suggests that individuals with higher levels of education tend to exhibit healthier lifestyles, better physical and mental health, lower morbidity rates, and increased life expectancy (12). This conclusion has been empirically validated in several developed countries. Studies from Canada, the United States (13), Denmark (14), and Sweden (15) consistently demonstrate a robust positive association between educational attainment and health status. Furthermore, research indicates that this relationship is not solely mediated by economic resources; socio-emotional skills, health literacy, information acquisition capabilities, and improved health-related decision-making also appear to be significant mediating factors (16).

As the world’s largest developing country, China offers a unique context for investigating the causal relationship between HE and health outcomes. Over the past three decades, China has undergone profound socioeconomic transformations, including significant expansion in access to HE and comprehensive reforms in its healthcare system (17). Since the late 1990s, China has implemented policies to expand higher education, raising the gross enrollment ratio from around 9.8% in 1998 to over 59.6% in 2022. Concurrently, public health indicators have significantly improved: life expectancy has increased from 67.8 years in 1981 to nearly 79 years in 2024 (18), and the infant mortality rate has dropped from over 37‰ in the early 1990s to 5.3‰ in 2022. Additionally, China is currently facing new challenges, such as a rapidly aging population (expected to account for over 30% of the total population by 2035) and an increasing burden of chronic non-communicable diseases (19). These dynamics highlight the need to understand the potential health implications of HE within the Chinese context, where educational attainment and health disparities remain closely intertwined.

While much academic attention has been devoted to the economic returns of HE—such as its effects on wages, employment, and occupational mobility—the non-economic benefits, particularly how HE environments shape long-term behavioral patterns affecting health, have received comparatively less scrutiny (20). Existing studies often find a positive association between educational attainment and health outcomes, indicating that better-educated individuals are generally healthier, experience lower rates of illness, and enjoy longer life expectancy (21). However, the magnitude, pathways, and causality of this relationship remain contested. Some scholars argue that the observed health benefits may be confounded by factors such as childhood socioeconomic status, parental education, or genetic endowments (22, 23). Others raise concerns about reverse causality, wherein individuals with better health are more likely to pursue HE (24). Moreover, while primary and secondary education have been widely studied, the specific health effects of HE as a distinct educational stage are less clearly documented. To address these gaps, this study uses data from the 2022 CFPS to examine the causal impact of HE on individual health outcomes, conceptualizing HE experience as an influential educational setting that shapes individual behaviors and life trajectories. Specifically, we employ self-rated health (SRH) and MH as the primary outcome variables, with HE attainment as the key explanatory variable. To ensure the robustness of our findings, we conduct several sensitivity checks, including alternative variable definitions, sample restrictions, and estimation using an ordered Probit model. To mitigate endogeneity concerns, we implement an instrumental variable (IV) strategy that leverages the interaction between China’s HE expansion policy and baseline regional enrollment rates. Furthermore, we investigate the mechanisms through which HE influences health outcomes, focusing on individual behaviors, family characteristics, and social factors. Lastly, we explore the intergenerational effects of parental HE on children’s health, shedding light on the broader social implications of educational attainment.

The marginal contributions of this study mainly focus on the following three aspects: First, by investigating the long-term health effects of HE, it expands the understanding of the non-economic returns to education. Second, using nationally representative 2022 CFPS data, we identify multiple channels—individual, familial, and societal—through which HE may affect both SRH and MH, providing robust empirical evidence from the Chinese context. Third, by considering both SRH and MH indicators, the study offers valuable insights for the development of integrated public health and education policies aimed at improving population health through educational investment.

The remainder of this paper is organized as follows: Section 2 reviews the relevant literature and presents the research hypotheses. Section 3 describes the data and methodology. Section 4 reports the empirical results and analysis. Section 5 concludes the study and provides policy recommendations.

## 2 Research hypotheses

This study hypothesizes that the mechanism by which HE affects health is multifaceted and operates at the individual, household, and societal levels.

At the individual level, individuals who have received HE tend to possess stronger cognitive abilities, which enable them to influence their health through improved health-related behaviors (25). People with HE typically exhibit greater health literacy, problem-solving skills, awareness of healthy behaviors, and risk aversion. As a result, they are more likely to engage in preventive health practices such as regular physical exercise, balanced nutrition, smoking cessation, and moderate alcohol consumption—behaviors that have been consistently associated with reduced morbidity, improved MH, and increased life expectancy (12). Moreover, highly educated individuals demonstrate superior capabilities in accessing medical resources, understanding health information, and adhering to medical advice (26).

At the household level, HE indirectly enhances individual health by increasing household income and improving the overall quality of life. HE is a key determinant of labor market outcomes, enabling individuals to secure higher-paying and more stable employment. This, in turn, boosts household income, which is a crucial factor in accessing healthcare services (27), nutritious food (28), and safer living environments (29)—all essential for maintaining good physical health (30, 31). Research indicates that higher household income is associated with reduced financial stress (32), which can alleviate the risk of MH disorders such as anxiety and depression (33, 34). In China, rapid economic development has been accompanied by growing income inequality, making the role of HE in elevating household income particularly prominent. Studies using data from the China Family Panel Studies (CFPS) have found that individuals with HE contribute to increased household income, which is subsequently associated with better health outcomes for themselves and their family members (35). Furthermore, educated individuals are more likely to marry partners with similar educational backgrounds, amplifying family resources and health benefits through assortative mating (36). This household-level pathway underscores the intergenerational and intra-household spillover effects of HE on health (37).

At the societal level, HE promotes health improvements through the enhancement of social capital, which refers to the networks, norms, and trust that facilitate collective action and resource sharing within communities. Educated individuals are more likely to participate in civic organizations, build diverse social networks, and engage in community activities—all of which strengthen their social capital (38). Social capital has been shown to positively influence health by providing emotional support, disseminating health-related information, and facilitating access to community resources (39). For example, individuals with strong social networks are more likely to receive timely health advice, adhere to treatment regimens, and adopt healthier lifestyles (40). In China, social networks (guanxi) play a central role in daily life, and HE can amplify these effects by enabling individuals to navigate social systems more effectively (41). Additionally, HE fosters a stronger sense of social responsibility and awareness of public health issues, encouraging behaviors that benefit not only individuals but also the broader community, such as participating in health campaigns or advocating for environmental improvements (42). This societal-level mechanism highlights the role of HE as a public good, extending health benefits from individuals to the wider population (43).

Based on the preceding literature review and theoretical analysis, this study proposes the following research hypotheses:

*Hypothesis 1 (H1)*: Higher education has a significant positive causal effect on improving individual health outcomes (including both physical and mental health).

*Hypothesis 2 (H2)*: Higher education promotes individual health, at least in part, by facilitating the adoption of healthier behaviors.

*Hypothesis 3 (H3)*: Higher education indirectly improves individual health by increasing household income.

*Hypothesis 4 (H4)*: Higher education enhances individual health by strengthening social capital.

## 3 Data and methodology

### 3.1 Data

This study utilizes data from the 2022 CFPS, a nationally representative, multidisciplinary, and longitudinal social survey project conducted by the Institute of Social Science Survey (ISSS) at Peking University (44). The CFPS collects comprehensive information on education, health, socioeconomic status, and demographic characteristics. It covers 25 provincial-level administrative regions across China, excluding Hong Kong, Macao, Taiwan, Xinjiang, Tibet, Qinghai, Ningxia, Hainan, and Inner Mongolia. The survey adopts a multi-stage stratified probability sampling design, with a target sample size of 16,000 households, enabling it to provide a detailed and representative depiction of China’s social landscape in terms of population, economy, education, and health (44). The quality of the CFPS data has been widely recognized in academic research. This study uses the samples of individuals aged 18 and above from the 2022 CFPS. After excluding the samples with missing key information in the 2022 CFPS, a data set containing nearly 19,677 individuals was formed.

### 3.2 Variable construction

#### 3.2.1 Dependent variables

Health serves as the dependent variable in this study. Since health is inherently unobservable, the choice of appropriate proxies is crucial. In social epidemiology, health is often interpreted narrowly as physical health, typically measured using medical indicators such as mortality and morbidity (45). However, according to the World Health Organization (WHO), health is “a state of complete physical, mental and social well-being and not merely the absence of disease or infirmity” (46). Therefore, this study adopts a multidimensional approach by including two measures: SRH and MH. SRH is a subjective assessment of one’s overall health status. While inherently subjective, SRH has been widely validated as a reliable and robust predictor of morbidity and mortality (47, 48). It has also been shown to outperform objective health indicators in some predictive contexts (49). Given the limitations of using clinical indicators like mortality in large-scale social surveys (50), SRH remains a widely accepted and practical measure of general health in sociological research. In CFPS, respondents were asked, “How would you rate your overall physical health?” with five response categories: 1 = “Unhealthy,” 2 = “Fair,” 3 = “Relatively Healthy,” 4 = “Healthy,” and 5 = “Very Healthy.” Higher scores indicate better perceived health status. In addition to SRH, MH is also considered a crucial dimension of individual health (51). With growing awareness in public health research, MH has become an essential component of overall health assessment (52). It influences not only emotional stability and life satisfaction, but also behavioral performance, social functioning, and physiological health. Empirical studies have shown that individuals with better MH are more capable of managing stress, maintaining interpersonal relationships, and sustaining physical well-being (53). In this study, MH is measured using an adapted version of the Center for Epidemiologic Studies Depression Scale (CES-D) (54), originally developed by Radloff. The CES-D8 used here includes eight items such as: “I felt depressed,” “I found everything I did was an effort,” “My sleep was restless,” “I felt happy,” “I felt lonely,” “I enjoyed life,” “I felt sad,” and “I felt that life was not worth living.” Respondents rated the frequency of these experiences over the past week on a 4-point scale: 1 = “Rarely or none of the time (less than 1 day),” 2 = “Some or a little of the time (1–2 days),” 3 = “Occasionally or a moderate amount of time (3–4 days),” and 4 = “Most or all of the time (5–7 days).” For the two positively worded items, reverse scoring was applied. The total CES-D8 score ranges from 8 to 32, with higher scores indicating higher levels of depressive symptoms (i.e., poorer MH), and lower scores reflecting better MH.

#### 3.2.2 Independent variable

The independent variable in this study is HE, which represents whether an individual has received HE (55). Based on the CFPS question “What is the highest level of education you have completed?,” responses were categorized as follows: individuals who reported “junior college (associate degree),” “bachelor’s degree,” “master’s degree,” or “doctoral degree” were coded as 1 (has received HE); all other educational levels, including primary school, middle school, high school, and vocational high school, were coded as 0 (not received HE).

#### 3.2.3 Mediating variables

To explore the mechanisms through which HE may influence health, we include three mediating variables: health behaviors (56), household income (37) and social capital (41). Health behaviors are constructed based on four behavioral indicators: (1) smoking in the past month, (2) alcohol consumption at least three times per week, (3) regular napping, and (4) frequency of physical exercise. Each behavior is assigned a binary score: unhealthy behaviors (e.g., smoking, frequent drinking, lack of exercise, and no napping) are coded as 0, while healthy behaviors are coded as 1. The sum of these items yields a composite index of healthy behaviors ranging from 0 to 4, where higher scores reflect healthier lifestyles. Household income is measured by the natural logarithm of annual household income. Social capital is peroxided by the natural logarithm of annual expenditure on social gifts and interactions, as reported by the household.

#### 3.2.4 Control variables

To account for potential confounding factors that may affect both education and health, several control variables are included in the analysis: age (57), gender (53), household size (58), residence (59), marital status (36), and agricultural work (60). A complete list of control variables is provided in Table 1.

**Table 1:** Definition of variables.

Variable type	Variable	Definition
DV	MH	A composite depression index (CES-D8) was constructed based on responses to the following eight items: “I feel depressed,” “Everything I do is an effort,” “My sleep is restless,” “I feel happy,” “I feel lonely,” “I enjoy life,” “I feel sad,” and “I feel that life is not worth living.”
DV	SRH	“Unhealthy” = 1, “Fair” = 2, “Relatively healthy” = 3, “Healthy” = 4, and “Very healthy” = 5.
IV	HE	Individuals with college diploma, bachelor’s degree, master’s degree, or doctoral degree were coded as 1; others were coded as 0.
CV	Age	Age was calculated as 2022 minus the respondent’s birth year.
CV	Gender	Male = 1; Female = 0.
CV	Household size	Number of individuals living in the household.
CV	Residence	Urban or non-agricultural Hukou = 1; Agricultural Hukou = 0.
CV	Marital status	Currently cohabiting or married = 1; unmarried, divorced, or widowed = 0.
CV	Agricultural work	Agricultural work = 1; Non-agricultural work = 0.
MV	Health behaviors	A composite index of healthy behaviors was created based on four items: Smoking status, drinking alcohol more than three times per week in the past month, Taking midday naps, and Engaging in physical exercise.
MV	Household income	Taking the logarithm of annual household income.
MV	Social capital	Taking the logarithm of household gift expenditures in the past year.

### 3.3 Descriptive statistics

After excluding observations with missing or invalid responses, a final sample of 19,677 individuals was retained for analysis using Stata 17.0. Descriptive statistics for the main variables are presented in Table 2.

**Table 2:** Descriptive statistics of variables.

Variable	*N*	Mean	SD	Min	Median	Max
SRH	19,677	2.88	1.17	1	3	5
MH	19,677	13.83	4.13	8	13	32
HE	19,677	0.19	0.39	0	0	1
Age	19,677	46.21	16.16	18	46	97
Gender	19,677	0.5	0.5	0	1	1
Household size	19,677	4.06	2	1	4	16
Residence	19,677	0.29	0.46	0	0	1
Marital status	19,677	0.78	0.41	0	1	1
Agricultural work	19,677	0.43	0.5	0	0	1
Health behaviors	19,677	2.64	0.99	0	3	4
Household income	19,677	11.27	1.13	0	11.36	16.59
Social capital	19,677	6.86	2.9	0	7.6	12.21

### 3.4 Model specification

To estimate the impact of HE on health, we first establish an ordinary least squares (OLS) regression model, as shown in Equation 1:


(1) $$H_{\mathrm{ijk}}=\beta_{0}+\beta_{1}HE_{\mathrm{ijk}}+X_{\mathrm{ijk}}+\varphi_{\mathrm{ijk}}+\varepsilon_{\mathrm{ijk}}$$

In this study, H_ijk_ denotes the health status indicator of individual i, who resides in province j and was born in year k. This health status includes both SRH and MH, as discussed previously. HE_ijk_ represents the higher education experience of individual i in province j and birth year k. X_ijk_ is a vector of control variables accounting for individual and child characteristics that may influence health status, including age, gender, household size, residence, marital status and agricultural work. To account for regional disparities in healthcare resources and levels of economic development—which may affect both health status and educational attainment—this study incorporates the current province of residence as a regional fixed effect. φ_ijk_ denotes province-level fixed effects, while $\varepsilon_{\mathrm{ijk}}$ captures the random error term, which includes: (1) other unobserved or insignificant influencing factors; (2) measurement errors; and (3) random factors that are difficult to control or quantify.

## 4 Empirical results and analysis

### 4.1 Baseline regression

The baseline regression results are presented in Table 3. Columns (1) and (2) report the estimated effects of HE on SRH and MH, respectively, without controlling for fixed effects. Columns (3) and (4) show the corresponding estimates after including location fixed effects. The results indicate that HE has a significantly positive effect on SRH and a significantly negative effect on MH, both statistically significant at the 1% level.

**Table 3:** Results of baseline regression.

Variables	(1)	(2)	(3)	(4)
	SRH	MH	SRH	MH
HE	0.0858^***^ (0.0192)	−0.4606^***^ (0.0760)	0.0879^***^ (0.0193)	−0.4289^***^ (0.0763)
Age	0.0224^***^ (0.0006)	0.0121^***^ (0.0022)	0.0225^***^ (0.0006)	0.0143^***^ (0.0022)
Gender	−0.2087^***^ (0.0159)	−0.7632^***^ (0.0584)	−0.2124^***^ (0.0159)	−0.7774^***^ (0.0580)
Household size	−0.0075^*^ (0.0044)	−0.0309^*^ (0.0159)	−0.0115^**^ (0.0045)	−0.0609^***^ (0.0162)
Residence	0.0001 (0.0186)	−0.7021^***^ (0.0697)	−0.0149 (0.0191)	−0.6399^***^ (0.0715)
Marital status	−0.0443^**^ (0.0207)	−0.5616^***^ (0.0807)	−0.0335 (0.0208)	−0.5338^***^ (0.0809)
Agricultural work	−0.0387^**^ (0.0187)	0.2213^***^ (0.0676)	−0.0426^**^ (0.0191)	0.0872 (0.0690)
Constant	2.0192^***^ (0.0336)	14.4138^***^ (0.1286)	2.0192^***^ (0.0724)	14.0460^***^ (0.2730)
Province FE	NO	NO	YES	YES
Observations	19,677	19,677	19,677	19,677

^*^, ^**^, and ^***^ indicate the significance at the 10, 5, and 1% levels, respectively.

It is worth noting that in the SRH variable, higher scores denote better perceived health, while in the MH measure, higher scores indicate more severe depressive symptoms. Therefore, regardless of whether fixed effects are included, HE exerts a significant impact on both SRH and MH, with results consistently significant at the 1% level.

### 4.2 Robustness tests

#### 4.2.1 Alternative definition of explanatory variable

To further verify the reliability of our baseline regression results, we conduct a robustness test by redefining the key explanatory variable. In the baseline model, HE is defined as a binary variable equal to one if the respondent has attained at least a college diploma (i.e., associate degree or above). However, within China’s HE system, there are fundamental differences among associate, undergraduate, and postgraduate education in terms of educational objectives, training approaches, and socioeconomic returns (61). Specifically, associate programs are typically more vocational and skill-oriented, whereas undergraduate and postgraduate education place greater emphasis on comprehensive development and theoretical advancement. These qualitative distinctions suggest that different levels of HE may influence health outcomes through heterogeneous mechanisms. By aggregating associate, undergraduate, and postgraduate education into a single category, the baseline model implicitly assumes a homogeneous effect of all HE levels on health, which may not reflect the actual situation. To address this concern, we redefine the HE variable based on the survey question: “What is the highest level of education the respondent has completed?” Responses indicating attainment of a university bachelor’s degree, master’s degree, or doctoral degree are categorized as having received HE (coded as 1), while those with no formal education, primary, junior high, senior high, vocational high school, or associate degree are categorized as not having received HE (coded as 0). The regression results using this redefined variable remain consistent with the baseline findings (see Columns (1) and (2) of Table 4). Even with the revised classification, HE continues to exert a significant positive effect on both SRH and MH, with the results remaining significant at the 1% level.

**Table 4:** Alternative definition of the explanatory variable and sample adjustments.

Variables	(1)	(2)	(3)	(4)
	SRH	MH	SRH	MH
Alternative HE	0.1213^***^ (0.0229)	−0.2978^***^ (0.0932)		
HE			0.0725^***^ (0.0203)	−0.4881^***^ (0.0808)
Age	0.0224^***^ (0.0006)	0.0165^***^ (0.0021)	0.0229^***^ (0.0006)	0.0157*** (0.0023)
Gender	−0.2115^***^ (0.0159)	−0.7807^***^ (0.0580)	−0.2150^***^ (0.0167)	−0.7845^***^ (0.0605)
Household size	−0.0117^**^ (0.0045)	−0.0567^***^ (0.0162)	−0.0116^**^ (0.0047)	−0.0580^***^ (0.0167)
Residence	−0.0127 (0.0189)	−0.6881^***^ (0.0707)	−0.0110 (0.0201)	−0.5709^***^ (0.0752)
Marital status	−0.0329 (0.0208)	−0.5385^***^ (0.0809)	−0.0363^*^ (0.0218)	−0.5109^***^ (0.0847)
Agricultural work	−0.0443^**^ (0.0191)	0.1065 (0.0689)	−0.0390^**^ (0.0195)	0.0727 (0.0704)
Constant	2.0293^***^ (0.0720)	13.8690^***^ (0.2696)	1.9235^***^ (0.0441)	14.4684^***^ (0.1687)
Province FE	YES	YES	YES	YES
Observations	19,677	19,677	18,256	18,256

*, **, and *** indicate the significance at the 10, 5, and 1% levels, respectively.

#### 4.2.2 Exclusion of special samples

Among China’s provincial-level administrative divisions, Beijing, Tianjin, Shanghai, and Chongqing are four municipalities directly under the central government. These municipalities often exhibit distinct economic and political characteristics, with significantly higher levels of economic development, better access to HE resources, and more advanced healthcare systems compared to other regions (62). Such disparities may introduce upward bias in estimating the effect of HE on health, thereby affecting the accuracy of the overall estimation. To address this concern and test the robustness of our findings, we excluded these four municipalities from the sample and re-estimated the baseline model. The regression results, presented in columns (3) and (4) of Table 4, indicate that HE continues to exert a significantly positive effect on both SRH and MH. This suggests that our main findings are robust and not driven by region-specific sample characteristics.

#### 4.2.3 Alternative regression model

Given that the dependent variables in our analysis are ordinal categorical variables, applying a linear regression model (e.g., OLS) may not fully capture their discrete and ordered nature. Therefore, to further validate the robustness of our results, we employed an ordered Probit model, which is more appropriate for dealing with ordinal dependent variables. Table 5 reports the estimation results from the ordered Probit model. The findings remain consistent with those obtained from the baseline OLS model in terms of both the direction and significance of the effects of HE on health. This further confirms the robustness of our conclusions under alternative model specifications.

**Table 5:** Alternative regression model.

Variables	(1)	(2)
	SRH	MH
HE	0.0934^***^ (0.0188)	−0.0955^***^ (0.0197)
Age	0.0214^***^ (0.0006)	0.0025^***^ (0.0005)
Gender	−0.2047^***^ (0.0154)	−0.1978^***^ (0.0145)
Household size	−0.0111^**^ (0.0044)	−0.0133^***^ (0.0040)
Residence	−0.0085 (0.0185)	−0.1564^***^ (0.0180)
Marital status	−0.0248 (0.0202)	−0.1161^***^ (0.0196)
Agricultural work	−0.0446^**^ (0.0185)	0.0184 (0.0170)
Observations	19,677	19,677

*, **, and *** indicate the significance at the 10, 5, and 1% levels, respectively.

### 4.3 Endogeneity analysis

#### 4.3.1 Construction of instrumental variables

In the baseline regression analysis presented earlier, we found that HE has a significant positive effect on individual health outcomes. However, this relationship may be subject to endogeneity issues, which primarily arise from two sources. First, an individual’s health status may influence their ability and willingness to pursue HE, leading to reverse causality (24). Second, unobserved individual characteristics—such as family background and cognitive ability—may simultaneously affect both education attainment and health outcomes (62, 63), resulting in omitted variable bias. To address these potential endogeneity concerns, we employ the Two-Stage Least Squares (2SLS) method for endogeneity testing. This method requires finding an instrumental variable that satisfies two key assumptions for causal inference: (1) The instrumental variable must be highly correlated with the endogenous explanatory variable. (2) The instrumental variable must be uncorrelated with the error term, meaning it cannot affect the dependent variable through other channels but only through the endogenous variable (64).

The implementation of the 1999 HE expansion policy in China provides a plausible exogenous shock for constructing an IV. On one hand, the policy led to a dramatic increase in university admissions—from 1.08 million in 1998 to 1.6 million in 1999—representing a 48% growth rate. This sharp increase suggests a strong correlation between the policy and individuals’ opportunities to access HE (65). On the other hand, the policy was introduced with urgency and was largely unanticipated. In response to the 1997 Asian Financial Crisis and the employment challenges caused by the 1998 state-owned enterprise reforms, the National Development and Reform Commission and the Ministry of Education issued an emergency directive in 1999 mandating a significant expansion in university enrollment. Due to the abrupt and large-scale nature of this policy, it is unlikely that individuals could have influenced its implementation through personal choices, thereby supporting the exogeneity of the instrument. Notably, the 1999 HE expansion policy has been widely used in prior studies as a valid instrument for estimating returns to education and intergenerational mobility (66).

Nevertheless, using the expansion policy alone as an instrument may be insufficient to fully capture regional variations in HE levels. Therefore, to enhance the relevance of our instrument, we utilized the regional heterogeneity of higher education (HE) before the policy was implemented. Based on earlier research, we interacted the post-policy cohort indicators with the initial provincial HE enrollment levels to form our instrumental variable. The rationale is that regions with higher initial HE levels are better able to absorb the enrollment rate growth caused by the expansion policy, thereby generating greater exogenous differences in the relevant cohorts (67). we construct an interaction term between the policy shock and the initial regional level of HE as our IV. The specific construction method is as shown in Equation 2:


(2) $$\mathrm{IV}=\mathrm{pos}t_{1999}*\mathrm{ih}e_{1998}$$

**Figure 1:**
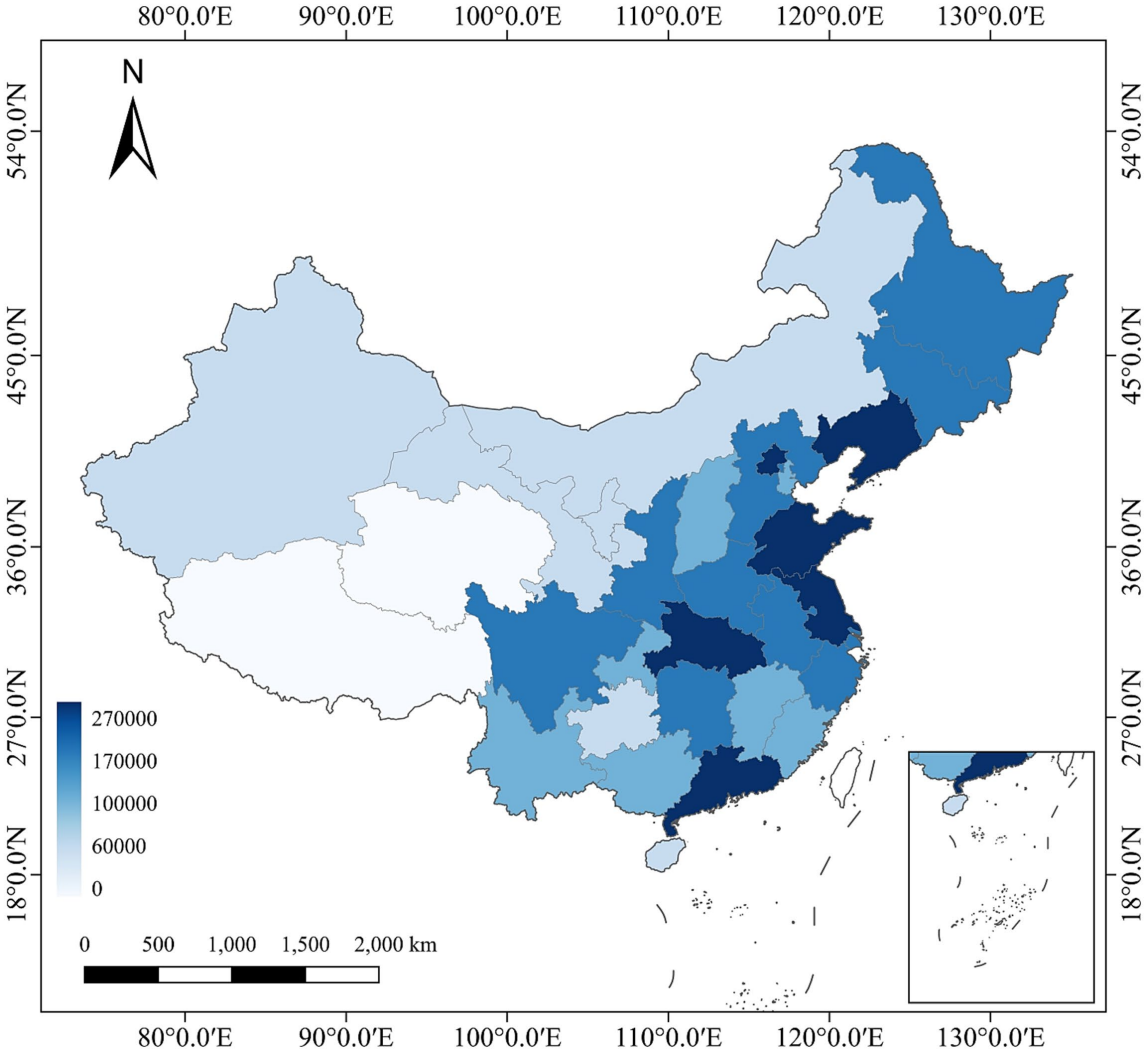
Number of students enrolled in HE by Province in 1998.

The variable post_1999_ represents the treatment group affected by the expansion policy. According to Chinese education law regarding school enrollment age, the earliest cohort affected by the policy would be those born in or after 1981. Thus, individuals born in 1981 or later are assigned a value of 1, and 0 otherwise. The variable Ihe_1998_ denotes the initial level of HE in each province, measured by the number of HE students enrolled in 1998 (Figure 1).

#### 4.3.2 Results of the instrumental variable approach

The first-stage regression results, reported in Table 6, show an F-statistic of 148.44, far exceeding the conventional threshold of 10, indicating a strong correlation between the instrument and the endogenous explanatory variable. Furthermore, the Kleibergen-Paap rk Wald F statistics (KP-Wald F statistics) are 405.966 and 295.785, respectively, which are well above the Stock-Yogo critical values, thereby ruling out the weak instrument problem. Overall, these tests confirm the validity of using the 1999 HE expansion policy and its interaction with initial regional educational levels as IV. The endogeneity test provides further support for Hypothesis 1 and demonstrates the robustness and reliability of the 2SLS estimation results based on the constructed instruments.

**Table 6:** Instrumental variable method test results.

Variables	(1)	(2)	(3)
	Phase I	Phase 2	
	HE	SRH	MH
IV	0.0119^***^ (0.0006)		
HE		0.5764^***^ (0.1497)	−1.4747^***^ (0.5621)
Age	−0.0045^***^ (0.0002)	0.0267^***^ (0.0014)	0.0034 (0.0053)
Gender	0.0029 (0.0049)	−0.2146^***^ (0.0161)	−0.7581^***^ (0.0586)
Household size	−0.0194^***^ (0.0013)	−0.0027 (0.0053)	−0.0495^***^ (0.0189)
Residence	0.2030^***^ (0.0071)	−0.1131^***^ (0.0352)	−0.4963^***^ (0.1329)
Marital status	0.0187^***^ (0.0070)	−0.0411^*^ (0.0213)	−0.5441^***^ (0.0816)
Agricultural work	−0.0649^***^ (0.0052)	−0.0075 (0.0220)	0.1408^*^ (0.0811)
Constant	0.3973^***^ (0.0324)	1.6700^***^ (0.1298)	15.0437^***^ (0.3736)
F statistics	148.44		
KP-Wald F statistics		405.966	295.785
Observations	19,677	19,677	19,677

*, **, and *** indicate the significance at the 10, 5, and 1% levels, respectively.

### 4.4 Mechanism analysis

The aforementioned studies have fully demonstrated a significant impact of HE on health. It is therefore essential to delve deeper into the intrinsic mechanisms through which HE affects health. This study will select corresponding mediating variables at the individual, family, and societal levels and employ mediation models to conduct mechanism analysis (68), the specific construction methods are as shown in Equations 3 and 4.


(3) $$\begin{array}{c} M_{\mathrm{ijk}}=\alpha_{0}+\alpha_{1}HE_{\mathrm{ijk}}+X_{\mathrm{ijk}}+\varphi_{\mathrm{ijk}}+\varepsilon_{\mathrm{ijk}} \end{array}$$

(4) $$\begin{array}{c} H_{\mathrm{ijk}}=\gamma_{0}+\gamma_{1}HE_{\mathrm{ijk}}+\theta M_{\mathrm{ijk}}+X_{\mathrm{ijk}}+\varphi_{\mathrm{ijk}}+\varepsilon_{\mathrm{ijk}} \end{array}$$

In the model, M_ijk_ denotes the mediating variables, including health behavior (56), Household income (37), and social capital (41). Health behavior is constructed from four questions: “Did you smoke in the past month?,” “Do you drink alcohol more than three times a week?,” “Do you have a nap habit?,” and “Exercise frequency.” For these four questions, unhealthy behaviors (smoking, drinking >3/week, no nap, no exercise) are each assigned a value of 0, while healthy behaviors (no smoking, no drinking, napping, having physical exercise) are each assigned a value of 1. The sum score of these four questions serves as a unified health behavior index, with a maximum score of 4 and a minimum score of 0. A higher score indicates healthier daily behavior, while a lower score indicates unhealthier daily behavior. Household income is represented by the log-transformed total annual household income. Social capital is represented by the log-transformed annual expenditure on social interactions/networking. H_ijk_ denotes the health status indicator for individual i residing in province j and born in year k, including the aforementioned SRH and MH. HE_ijk_ indicates whether the individual has received HE. X_ijk_ represents control variables, including age, gender, household size, residence, marital status, and agricultural work, etc. denotes location fixed effects.

Table 7 presents the results of the mediation analysis with health behaviors as the mediating variable. Column (1) shows that HE significantly influences health behaviors when health behavior is treated as the mediator. Columns (2, 3) examine the effects of both the independent variable (HE) and the mediator (health behaviors) on SRH and MH outcomes, respectively. The results indicate that health behaviors have a significantly positive effect on SRH, but a significantly negative effect on MH. These findings suggest that HE improves both SRH and MH through its positive impact on health behaviors, thereby supporting Hypothesis 2. Table 8 reports the mediation analysis results with household income as the mediating variable, while Table 9 presents the results using social capital as the mediator. In both cases, HE significantly influences the mediating variables—household income and social capital. Furthermore, both household income and social capital exert a significantly positive effect on SRH and a significantly negative effect on MH. These findings imply that HE enhances health outcomes—both SRH and MH—by increasing household income and social capital, thereby providing empirical support for Hypotheses 3 and 4.

**Table 7:** Test of health behavior mechanism.

Variables	(1)	(2)	(3)
	Health behaviors	SRH	MH
Health behaviors		0.0501^***^ (0.0089)	−0.4311^***^ (0.0322)
HE	0.4312^***^ (0.0180)	0.0880^***^ (0.0197)	−0.2430^***^ (0.0771)
Constant	2.9919^***^ (0.0619)	2.0196^***^ (0.0777)	15.3357^***^ (0.2890)
Control	YES	YES	YES
Province FE	YES	YES	YES
Observations	19,677	19,677	19,677

*, **, and *** indicate the significance at the 10, 5, and 1% levels, respectively.

**Table 8:** Test of household income mechanism.

Variables	(1)	(2)	(3)
	Household income	SRH	MH
Household income		0.0496^***^ (0.0083)	−0.2807^***^ (0.0350)
HE	0.3929^***^ (0.0183)	0.1074^***^ (0.0196)	−0.3186^***^ (0.0773)
Constant	11.1746^***^ (0.0708)	2.5729^***^ (0.1169)	17.1827^***^ (0.4750)
Control	YES	YES	YES
Province FE	YES	YES	YES
Observations	19,677	19,677	19,677

*, **, and *** indicate the significance at the 10, 5, and 1% levels, respectively.

**Table 9:** Test of social capital mechanism.

Variables	(1)	(2)	(3)
	Social capital	SRH	MH
Social capital		0.0074^***^ (0.0028)	−0.0433^***^ (0.0107)
HE	0.3938^***^ (0.0567)	0.0909^***^ (0.0193)	−0.4118^***^ (0.0764)
Constant	4.8591^***^ (0.2600)	2.0554^***^ (0.0742)	14.2565^***^ (0.2792)
Control	YES	YES	YES
Province FE	YES	YES	YES
Observations	19,677	19,677	19,677

*, **, and *** indicate the significance at the 10, 5, and 1% levels, respectively.

### 4.5 Intergenerational impact of HE

In the preceding sections, we have demonstrated through baseline regressions and robustness tests that HE has a significant positive effect on individual health. However, the influence of education extends beyond the individual level—it may also exert a profound intergenerational impact on the health of the next generation through family-based transmission mechanisms (69). Specifically, parents with HE tend to possess greater health awareness and are more likely to adopt healthy lifestyles—such as maintaining a balanced diet, following regular sleep patterns, and limiting tobacco and alcohol consumption (70). These behaviors are often passed down to their children, thereby improving the children’s health outcomes. Moreover, higher educational attainment is typically associated with enhanced economic capacity and access to resources, enabling parents to provide better living conditions and healthcare services for their children, such as access to higher-quality medical care, nutritious food, and a healthier living environment. In addition, highly educated parents are more likely to make long-term investments in their children’s education and health (71). With greater foresight and planning, they focus on their children’s nutrition, physical activity, and mental development from an early age, laying a solid foundation for long-term health. To empirically examine the impact of parental HE on children’s health, we constructed regression models such as Equations 5 and 6:


(5) $$\begin{array}{c} H_{\mathrm{ijk}}=\beta_{0}+\beta_{1}\mathrm{MH}E_{\mathrm{ijk}}+X_{\mathrm{ijk}}+\varphi_{\mathrm{ijk}}+\varepsilon_{\mathrm{ijk}} \end{array}$$

(6) $$\begin{array}{c} H_{\mathrm{ijk}}=\beta_{0}+\beta_{1}\mathrm{FH}E_{\mathrm{ijk}}+X_{\mathrm{ijk}}+\varphi_{\mathrm{ijk}}+\varepsilon_{\mathrm{ijk}} \end{array}$$

MHE_ijk_ denotes whether the mother has received HE, and FHE_ijk_ denotes whether the father has received HE. Individuals with an associate degree, bachelor’s degree, master’s degree, or doctoral degree are coded as 1, while all other educational levels are coded as 0. X_ijk_ represents a set of control variables for individual and child-specific characteristics that may influence health outcomes, including gender, age, number of siblings, household registration (Hukou), and whether the individual is engaged in agricultural work. Table 10 presents the impact of parental HE on children’s health outcomes. The results indicate that the father’s HE level has a statistically significant effect on children’s SRH, with significance at least the 5% level. In contrast, the mother’s HE level has a significantly positive effect on children’s MH, statistically significant at the 1% level. This gender-differentiated impact likely stems from traditional family structures and gendered caregiving roles prevalent in the Chinese context. For the paternal influence, a father’s HE is strongly correlated with higher family socioeconomic status. This enhanced economic capacity translates into tangible resources that bolster a child’s SRH, such as improved nutrition, better housing conditions, and greater access to quality healthcare services. Thus, the father’s role, often culturally defined as the primary breadwinner, creates a crucial pathway from education to the child’s physical well-being via economic mechanisms (72). The significant influence of maternal HE on a child’s MH, however, points to a different, more direct nurturing mechanism. This finding can be interpreted through several gender-based theoretical lenses: First, the maternal time and caregiving investment theory suggests that mothers, particularly in East Asian societies, are traditionally the primary caregivers. A mother with higher education may not only spend time with her child but also invest this time more effectively (73). Her education can foster advanced parenting skills, greater emotional intelligence, and an increased ability to recognize and respond to a child’s psychological needs and distress signals. This high-quality interaction is fundamental for healthy socio-emotional development. Second, higher education significantly enhances a mother’s health literacy. An educated mother is better equipped to acquire, process, and understand health information, including complex knowledge about child psychology and mental well-being. This enables her to create a more stimulating and emotionally supportive home environment, actively fostering resilience and positive coping mechanisms in her children.

**Table 10:** Intergenerational impact of HE.

Variables	(1)	(2)	(3)	(4)
	SRH	MH	SRH	MH
FHE	0.0477^**^ (0.0190)	−0.0140^*^ (0.0081)		
MHE			0.0158 (0.0897)	−0.0670^***^ (0.0143)
Age	0.0218^***^ (0.0006)	0.0179^***^ (0.0020)	0.0218^***^ (0.0006)	0.0178^***^ (0.0020)
Gender	−0.2122^***^ (0.0159)	−0.7792^***^ (0.0581)	−0.2120^***^ (0.0159)	−0.7782^***^ (0.0581)
Household size	−0.0131^***^ (0.0045)	−0.0532^***^ (0.0161)	−0.0131^***^ (0.0045)	−0.0535^***^ (0.0161)
Residence	0.0019 (0.0187)	−0.7258^***^ (0.0701)	0.0030 (0.0187)	−0.7178^***^ (0.0700)
Marital status	−0.0317 (0.0208)	−0.5406^***^ (0.0810)	−0.0322 (0.0208)	−0.5469^***^ (0.0812)
Agricultural work	−0.0489^**^ (0.0191)	0.1180^*^ (0.0688)	−0.0490^**^ (0.0191)	0.1174^*^ (0.0688)
Constant	2.0804^***^ (0.0711)	13.7400^***^ (0.2651)	2.0823^***^ (0.0710)	13.7484^***^ (0.2650)
Observations	19,677	19,677	19,677	19,677

*, **, and *** indicate the significance at the 10, 5, and 1% levels, respectively.

## 5 Conclusion and policy implications

Based on data from the 2022 CFPS, this study empirically examines the causal effects of HE on individual health, viewing HE as a pivotal educational setting influencing life-course health trajectories. The study first employed initial regression analysis to investigate the impact of HE on SRH and MH. The robustness of the findings was then verified through various checks, including using alternative explanatory variables, excluding specific samples, and changing models. To mitigate potential endogeneity issues, this paper further constructed an interaction term between the HE expansion policy and the initial regional HE level as an IV for estimation. Subsequently, the paper explored the transmission mechanisms through which HE affects individual health, focusing on how this educational experience shapes behaviors and accesses resources at personal, family, and social levels. Finally, the study also investigated the intergenerational impact of parental HE on children’s health.

The main findings of this study are summarized as follows: (1) HE has a significant impact on both SRH and MH, at the 1% significance level. (2) The above conclusion remains valid after undergoing various robustness tests. (3) The significant impact of HE on individual health remains robust after addressing endogeneity issues using the IV method. (4) HE primarily enhances individual health through three mechanisms: improving health behaviors, increasing household income, and strengthening social capital. (5) Parental HE has a significant intergenerational impact on children’s health: father’s education level significantly affects children’s SRH (at least at the 5% significance level), and mother’s education level significantly affects children’s MH (at least at the 1% significance level).

Based on the above research findings, this paper proposes the following policy recommendations: (1) Continuously promote the popularization and equitable development of HE, improve educational accessibility, and narrow the education-health gap. HE not only brings economic returns but is also a crucial pathway to improving national health levels. The government should continue to increase investment in HE, especially tilting toward central and western regions and areas with weaker educational resources. Measures such as fiscal transfers, improving scholarship and grant systems, and supporting the development of regional universities should be implemented to enhance the accessibility of HE, thereby promoting educational equity and narrowing the health disparities between different regions and groups. (2) Strengthen health education and behavioral interventions and enhance the health literacy of university students. The study found that health behavior is an important mechanism through which HE influences health. It is recommended to integrate health education into the HE curriculum, offering courses such as health management, MH, and nutrition, to guide students in adopting healthy lifestyles. Concurrently, universities should actively organize health lectures, psychological counseling services, and sports activities to strengthen students’ health awareness and self-management abilities, laying a solid foundation for lifelong health. (3) Emphasize and support family education to promote healthy intergenerational transmission. Parental (especially maternal) HE has a significant intergenerational impact on children’s health, highlighting the importance of the family educational environment and the health-related behaviors modeled by educated parents. It is recommended to improve family support policies that encourage and support parents in enhancing their own educational attainment and health literacy, such as providing opportunities for continuing education and disseminating family health knowledge, to achieve a virtuous intergenerational cycle of education and health. (4) Address the dual disadvantage of education and health among vulnerable groups through coordinated intervention strategies. For groups facing a dual disadvantage in both education level and health status, a comprehensive “education + health” intervention strategy should be adopted. By providing re-education opportunities such as vocational training and adult education, and ensuring access to basic medical services, these strategies can help them break the “disadvantage cycle” where education and health mutually constrain each other, contributing to the goals of common prosperity and a Healthy China.

The limitations of this study are as follows, providing directions for future research: (1) Data Limitations: This study only utilizes cross-sectional data from 2022. Future research could use panel data with a longer time span and employ methods such as fixed effects models to more comprehensively examine the long-term dynamic impact of HE on health. (2) Endogeneity Issues: Although this paper used the IV method, potential endogeneity issues may not have been fully resolved. Future research could attempt to use other more effective econometric methods to further identify causal effects. (3) Cultural Heterogeneity: The mechanisms and effects of HE on health may differ across cultural backgrounds. Future research could expand the scope to other countries or regions to explore the role of cultural factors.

## Data Availability

Publicly available datasets were analyzed in this study. This data can be found at: the datasets presented in this study are publicly available via the CFPS database, http://www.isss.pku.edu.cn/cfps/.
